# Influence of Thermal Treatment Conditions and Fruit Batches Variability on the Rheology and Physicochemical Profile of Golden Delicious Apple Purée

**DOI:** 10.3390/foods14223912

**Published:** 2025-11-15

**Authors:** Shichao Li, Alessandro Zanchin, Anna Perbellini, Sebastiano Meggio, Nicola Gabardi, Marco Luzzini, Lorenzo Guerrini

**Affiliations:** 1 Department of Land, Environment, Agriculture and Forestry, University of Padova, 35020 Legnaro, Italy; shichao_li77@163.com (S.L.); anna.perbellini@unipd.it (A.P.); sebastiano.meggio@studenti.unipd.it (S.M.); lorenzo.guerrini@unipd.it (L.G.); 2 Independent Researcher, 38023 Cles, Italy

**Keywords:** food engineering, rheology, enzymes residual activity, syneresis, colour analysis

## Abstract

Apple purée is a processed food typically obtained from ground apples, where quality depends on colour, consistency, and shelf-life. Thermal treatments are commonly applied to adjust rheology and deactivate enzymes responsible for post-packaging deterioration. This study evaluated the effects of heating temperature (87–102 °C) and duration (6–17 min) on the physical and chemical properties of Golden Delicious apple purée. Three independent batches were processed to examine intra-varietal variability. Chemical analyses assessed enzyme activity and nutritional profile, while physical tests focused on rheology. Image analysis was employed to characterise colour and syneresis. Results showed that short-duration heating at higher temperatures (>100 °C, <12 min) achieved desirable rheological properties but intensified browning. No significant correlations were found between residual enzymatic activity, polyphenol content, antioxidant activity, and thermal treatment conditions. This suggests that changes in colour and texture are primarily related to the physical parameters of heating independently of the origin batch. In contrast, the batch had a significant impact on enzymatic and nutritional profiles, highlighting the need for strict monitoring of incoming fruit. Overall, the heating conditions influenced the visual and textural quality of the purée, while the variability in raw materials remained a significant factor affecting its biochemical characteristics.

## 1. Introduction

Apple is one of the most widely cultivated fruit crops globally and holds significant importance in the Mediterranean region. For example, approximately 13% of Italy’s apple production is directed toward the food industry, with apple juice being the primary product derived from apple processing. Apple purée, another valuable product, is typically produced from apples deemed unsuitable for direct sale, thus playing a crucial role in the sustainability of the apple sector [1].

The production of apple purée involves grinding and cooking fresh apples to create a homogeneous suspension of fruit particles in the apple juice. The grinding process is crucial for achieving a uniform particle distribution, which is essential for maintaining the consistency of the purée [2]. Cooking serves two main purposes: enhancing the texture of the purée and preserving the product from microbiological and physical deterioration [3]. An interaction between grinding configuration and heating temperature was also observed, affecting the quality of the final product [2]. Pasteurisation, typically conducted at temperatures between 57 °C and 72 °C, effectively eliminates harmful fungi and bacteria. However, residual endogenous enzymes can persist and contribute to purée degradation, including after the packaging stage [4]. The most relevant enzymes in fruit purées are pectin methylesterase (PME) [4], polyphenol oxidases (PPOs) [5], and peroxidases (POs) [6]. PME hydrolyses the methyl ester bonds in cell wall pectin, leading to a deterioration in texture in apple-based products. This process can result in clump formation, water release (known as syneresis), and a loss of consistency [2,7]. On the contrary, pectin preservation and solubilisation are pursued to enhance the rheological properties of purées [8]. PPOs catalyse the hydroxylation of phenolic compounds in the presence of oxygen, while POs decompose hydrogen peroxide into water and oxygen [9,10]. Both PPOs and POs are responsible for “enzymatic browning” in processed fruit foods, which can degrade polyphenols and phenols, or rather pigments like anthocyanins, leading to colour changes and potentially affecting the appearance of the purée [11,12]. Fruit processing under oxygen-free conditions is a viable solution for preventing the enzymatic browning [13]. The total phenols content (TPC) is valued for the nutritional profile of apples; on the other hand, high TPC may cause intense purée colour and increased browning risk. Antioxidant compounds, such as ascorbic acid (vitamin C), are naturally found in apples and can prevent enzymatic oxidation. Consequently, the flavour and taste of puréed foods might be inferior to those of fresh products due to the activity of these enzymes.

Texture and colour are critical quality attributes in apple purée [14,15], with viscosity serving as a key marker for consumer preference [16], while syneresis is generally undesirable [14]. For this reason, reaching a target range of values is essential from the production side [17]. Since enzyme activity can persist post-packaging, researchers have explored various methods and preservatives to prevent enzymatic deterioration [18]. While chemical additives can manage these issues [11,19], increasing demand for healthier, preservative-free foods is prompting the food industry to explore physical treatments. High-temperature and high-pressure treatments are among the most studied processes [20,21]. In some cases, a temperature higher than 50 °C can deactivate PPOs [22]. Temperatures above 70–80 °C can significantly reduce enzyme activity and enhance texture by increasing consistency and reducing syneresis [23,24]. However, heating can also affect the product’s colour due to sugar caramelisation and Maillard reactions, leading to “non-enzymatic” browning [19], which can alter the original colour, flavour, and nutrient composition of fruit-based foods and impact consumer preferences [25,26,27,28].

Previous studies investigating the combined effects of temperature, pressure, and processing time on strawberry [29] and pineapple [30] purées have shown that temperature and time are key drivers of browning and polyphenol degradation, particularly in pineapple. Moreover, sufficient enzyme deactivation generally requires temperatures above 60 °C and high-pressure conditions (600 MPa are suggested). Among the process parameters in fruit-based products, heating temperature and duration are the most extensively studied [31,32]. Temperature is expected to promote non-enzymatic browning by accelerating several chemical reactions, while prolonged mixing at processing temperature may enhance purée consistency by solubilising polysaccharides. For example, temperatures above 70 °C promote the production of anthocyanins and other polyphenols [33]. Conversely, higher temperatures can favour polysaccharide gelation, further contributing to consistency.

This study aims to investigate the complex interplay between thermal treatment conditions and the physical and chemical properties of apple purées under controlled laboratory settings. Despite the recognised importance of rheological behaviour for process optimisation in the food industry [24,34] and its influence on consumer acceptance [35], current literature lacks detailed insights into how heating temperature and duration jointly affect the rheological characteristics of apple purées. This work addresses this gap by evaluating the effects of temperature, time, and their interaction on purée quality, while also considering intra-cultivar variability by testing three independent batches of apples. Response Surface Methodology (RSM) was applied as a statistical tool to minimise experimental effort and to predict a broader range of potential industrial processing conditions.

## 2. Materials and Methods

### 2.1. Sample Preparation

Apples ( *Malus domestica* var. Golden Delicious) were provided in 2024 by a local farming company in Val di Non (Trento, Italy) and stored at 4 °C for approximately five months. Samples were taken from three batches, namely, B17, B18, and B19. Batches originating from different areas within the province, which may have influenced their ripening stage, cultivation method, or rather, the nutrient content and the enzymatic activity. Before processing, apples were equilibrated to room temperature (15–18 °C), then they were manually peeled, cored, washed with fresh tap water, and sliced. A total of 1.8 kg of edible pulp was transferred into a HotmixPRO 5 Stars (Hotmix Pro, Modena, Italy) food processor, which simultaneously grinds or mixes while heating. On average, peels, cores, and pedicels accounted for 27% of the initial apple mass. Samples were prepared immediately before processing to minimise oxidation. Ascorbic acid (400 mg kg^−1^) was added prior to grinding at a fixed dose of 400 mg kg^−1^. Grinding lasted 15 min at 3000 rpm. Then, the blades were replaced with a mixing tool (300 rpm). Thermal treatment started once the purée reached the target temperature and lasted from 6 min 20 s to 17 min 42 s, as reported in Table 1. One purée per batch was left untreated as “Control”.

### 2.2. Experimental Design and Statistical Analysis

The effects of heating temperature (Temp) and time (Time) were tested using RSM, following a Central Composite Design (CCD) with five levels for each factor and 12 total combinations, including four replicates of the central point (12 min, 95 °C). Experiments were replicated across the three batches, forming a block design to enhance statistical robustness and repeatability. The heating temperatures were randomised to avoid confounding from batch variation and trial sequence (i.e., cooldown and warmup between consecutive trials).

Apple purées were analysed to correlate chemical and physical properties such as colour, density, viscosity, total soluble solids (TSSs), syneresis, pH, water content (WC), total polyphenol content (TPC), antioxidant capacity (TEAC), and residual enzymatic activities of POs, PPOs, and PME, with the thermal treatment parameters. The statistical analysis was based on the RSM, applied using the “rsm” package in RStudio (version 2024.12.1+563). Analysis of variance (ANOVA) was used to assess model significance (*p* < 0.05), while models with a lack-of-fit *p* > 0.05 were considered acceptable. In those cases where an RSM model was deemed unaffordable due to batch effects and interactions between the batch and the factors, a specific mixed model that included the batch effect was established.

### 2.3. Physical Analysis

The colour analysis was performed immediately after purée production, while the remaining physical analyses were carried out after the purée had cooled.

#### 2.3.1. Colour Analysis

A sample of hot purée was poured into three 100 mm diameter Petri dishes. After 30 min, an RGB photo was acquired from each dish (three repetitions per trial) using a Canon R10 camera (Canon Inc., Tokyo, Japan) at fixed light conditions from a black box. All images were segmented through Food-ColorInspector (http://www.cofilab.com accessed in November 2022), and the purées’ colours were assessed based on CIELab colour space. The purées’ colour after thermal treatment was evaluated as the distance from the colour of the control purée. Colour distance (ΔE) was computed according to Equation (1). Furthermore, Chroma and Hue describe the colour intensity and tone, respectively, according to Equations 2 and 3. Finally, the browning index (BI) was calculated with Equation (4), which is widely used for evaluating the colour deterioration on food and beverages [36].

(1) $$∆E\;=\;\sqrt{{\left(L_{c}^{*}-L_{i}^{*}\right)}^{2}+{\left(a_{c}^{*}-a_{i}^{*}\right)}^{2}+{\left(b_{c}^{*}-b_{i}^{*}\right)}^{2}\;}$$

(2) $$Chroma\;=\;\sqrt{a^{*2}\;+\;b^{*2}}$$

(3) $$Hue\;=\;{tan}^{-1}\left(\frac{b^{*}}{a^{*}}\right)$$

(4) $$BI\;=\;100\;*\;\frac{x-0.31}{0.172}\;,\;\;x\;=\;\frac{a\;+\;1.75L^{*}}{5.645L^{*}\;+\;a^{*}\;-\;3.012b^{*}}$$

where L* is the lightness value, a* is the red-green tonality, b* is the yellow-blue tonality, and c and i mean control and trial underwent thermal treatment purées, respectively (ΔE).

#### 2.3.2. Consistency Determination

The rheological properties of the apple purées were determined using an Anton Paar ViscoQC 300L rheometer (Anton Paar, Graz, Austria) equipped with a specific cylinder (L4) measuring 3 mm in radius (Rs) and 30 mm in length (ℓ), along with a 600 mL beaker of 45 mm radius (Rc). Apples were ground and cooked at 95 °C for 12 min to produce the test purée. Shear rates ($\dot{\gamma }$) and shear stress (τ) of the purée were computed at 28 °C across a range of rotation speeds from 10 to 150 rpm by recording the torque (M) after 60 s since the cylinder started rotating, following Equations (5) and (6). Rotation speed was converted into angular velocity (ω). Since fruit purées are typically non-Newtonian fluids, the consistency index (K) was calculated using Equation (7), where *n* represents the flow behaviour index. Finally, the flow behaviour index *n* was determined by computing the slope of the linear regression model obtained from the plot of log($\dot{\gamma }$) versus log(τ).

(5) $$\dot{\gamma }\;=\;\frac{2\;*\;\omega \;*\;{Rc}^{2}}{\left({Rc}^{2}\;-\;{Rs}^{2}\right)}$$

(6) $$\tau \;=\;\frac{M}{2\;*\;\pi \;*\;{Rs}^{2}\;*\;l}$$

(7) $$K\;=\;\frac{\tau }{\gamma^{n}}$$

The same rheometer was used to assess the consistency of specific purée samples. The consistency was measured at 50 rpm after 60 s of rotation. Fifty rpm was evaluated as the suitable torque and viscosity range rotation speed. The consistency was directly retrieved by the rheometer (mPa s). Three samples of 500 mL each were evaluated from each trial (three repetitions) at temperatures between 35 °C and 40 °C.

#### 2.3.3. Syneresis Determination

Image analysis was chosen for syneresis determination. After cooling (room temperature), 80.0 g of purée was poured into a metal cylinder 60 mm * 50 mm, diameter and height. Three measurements were conducted for each sample, and the results were averaged. Each cylinder is laid on coarse rectangular paper measuring 210 mm * 297 mm, with a density of 110 g m^2^. Papers were placed above regular wooden boards and on a levelled surface. After 24 h, fluid diffusion was detectable outside the cylinder. The cylinder containing the purée was removed, and an RGB image of each board was acquired from the black box at a fixed camera and target distance. The photos were manually cropped following the standard reference printed on the paper. The spot caused by the fluid diffusion from the metal cylinder was automatically segmented with the cloud service Arivis Cloud (https://www.arivis.cloud/ accessed in June 2024). Arivis Cloud returned a mask with which the syneresis was assessed as the fraction area occupied by the labelled spot on the whole paper. A higher area fraction means more fluid diffusion, hence more syneresis. The visual assessment was chosen instead of a common centrifugation because it returned results that were much closer to the real behaviour of standard purées after packaging. Moreover, the spot dimension has a higher impact on consumers or evaluation panellists [37,38].

#### 2.3.4. Water Content, pH and Total Soluble Solids

The WC of the purées was determined by oven drying at 105 °C for 24 h. For TSSs and pH measurements, filtered apple juice was required. Approximately 200 g of hot purée was filtered using a disc paper filter (85 g m^−2^, thickness: 180 µm, retention: 10–13 µm). TSSs were then measured in the filtrate using a digital refractometer (HI96801, HANNA Instruments, Villafranca Padovana, Italy ), while pH was determined with a digital pH metre (pH 70 Vio, XS Instruments, Carpi, Italy ).

### 2.4. Enzymatic Assays

Apple juice and specific enzyme extraction requested for the enzymatic assays were necessarily prepared immediately after purée production and frozen at −18 °C on the same day. However, the enzymatic assays were performed days after, aware that enzyme activity was still unaffected until four hours after thawing [19].

#### 2.4.1. Pectin Methylesterase Assay

Pure apple juice was filtered as described in 2.3.4 and used for this assay. PME residual activity was evaluated by titration of free carboxyl groups [39]. The reagents and methodology followed a standard methodology adapted for fruit purées [40]. A 1.0% (*w*/*v*) pectin solution with 0.3 M of sodium chloride (NaCl) was prepared, and the pH was raised to 7.5 with a sodium hydroxide (NaOH) 0.1 M solution. The reaction started when 5.0 mL of apple juice was added to 50.0 mL of pectin solution. NaOH 0.02 M was used as a titration solution to maintain a pH of around 7.5. The manual titration lasted 30 min at a constant 30 °C. PME was assessed as units mL^−1^ of sample. Unit means the moles of NaOH consumed in one minute. Moreover, a blank titration was performed daily on the pectin solution to quantify the carboxyl group releases or the carbonic acid (H_2_CO_3_) generation into water [41].

#### 2.4.2. Peroxidases Assay

First, enzyme extraction was performed with a specific extraction solution made of 4% (*w*/*v*) of bovine serum albumin, 1% (*v*/*v*) of Triton X-100, and potassium phosphate (K_3_PO_4_) 0.04M to reach pH 5.0. Exactly 10.0 g of purée were weighed and added to 20.0 mL of extraction solution, stirred for two minutes at sixty rpm, and filtered after 10 min with paper filters (85 g m^−2^, thickness 180 µm, retention 10–13 µm). The spectrophotometric assay using ABTS [2,2′-azino-bis(3-ethylbenzthiazoline-6-sulfonic acid)] as the substrate was employed to quantify PO activity [19]. An aliquot of 0.5 mL of enzyme extract was added to 1.0 mL of K_3_PO_4_ buffer (pH 6.5) and 1.0 mL of ABTS solution (0.0091 M). The reaction was initiated by adding 0.5 mL of hydrogen peroxide (1.5% *v*/*v*) [23,29]. The mixture was briefly shaken, and absorbance readings were taken 5 s after the final addition. The reaction was monitored in a 10 mm cuvette for 10 min at 405 nm using a single-beam spectrophotometer Jenway 6300 (Jenway Ltd, Essex, UK). Enzyme activity was expressed as the change in absorbance per minute per gram of purée (ABS min^−1^ g^−1^). Each measurement was performed in triplicate, including the control purées.

#### 2.4.3. Polyphenol Oxidase Assay

The enzyme extraction followed the procedure explained in 2.4.2. Still, in this case, the extraction solution consisted of a phosphate buffer, made of sodium phosphate monobasic dihydrate (NaH_2_PO_4_ + 2H_2_O) 0.2 M and sodium phosphate dibasic dihydrate (Na_2_HPO_4_ + 2H_2_O) 0.2 M to maintain pH at 6.5, and +0.5% (*v*/*v*). The spectrophotometric assay using L-DOPA (L-3,4-dihydroxyphenylalanine) as the substrate was employed to quantify PPO activity [19]. An aliquot of 0.5 mL of enzyme extract was mixed with 2.5 mL of L-DOPA 0.0015 M [23,29]. The mixture was briefly shaken, and absorbance readings were taken 5 s after the L-DOPA addition. The reaction was monitored in a 10 mm cuvette for 10 min at 480 nm using a single-beam spectrophotometer. Enzyme activity was expressed as the change in absorbance per minute per gram of purée (ABS min^−1^ g^−1^). Each measurement was performed in triplicate, including the control purées.

### 2.5. Chemical Analysis

The antioxidant capacity (TEAC) and TPC were assessed. Both assays followed a standard methodology, with minimal modifications [42]. 1.5 g of apple purée was dissolved in 5.0 mL of water [43], mixed using a vortex, and then centrifuged at 3800 × *g* for 5 min. The liquid phase was drawn off, and centrifugation was repeated twice, with 5.0 mL of water added each time. Finally, the TPC and TEAC were tested in 15.0 mL of the aqueous extract.

#### 2.5.1. Total Phenol Content

The Folin–Ciocalteu method was used for TPC estimation as gallic acid equivalents (mg kg^−1^), and the absorbance of samples was measured with a UV–Visible spectrophotometer at 740 nm. Five solutions containing 10–100 mg L^−1^ of gallic acid were used for calibration. The extracts were mixed with 1 mL of Folin–Ciocalteu reagent, diluted ten times, and sodium carbonate at 75.0 g L^−1^. Absorbance was recorded after a 120 min incubation.

#### 2.5.2. Antioxidant Capacity

The ABTS^●+^ oxidant radical was generated by mixing ABTS at 0.007 M with potassium persulfate at 0.007 M, followed by 24 h of incubation. Subsequently, the ABTS^●+^ solution was stabilised at pH 7.0 using a phosphate buffer. Absorbance was measured at 730 nm after combining 0.2 mL of apple extract with 2.0 mL of diluted ABTS^●+^. A calibration curve was prepared with six Trolox solutions ranging from 3.120 mg L^−1^ to 50.000 mg L^−1^, diluted in phosphate buffer.

## 3. Results

**Figure 1:**
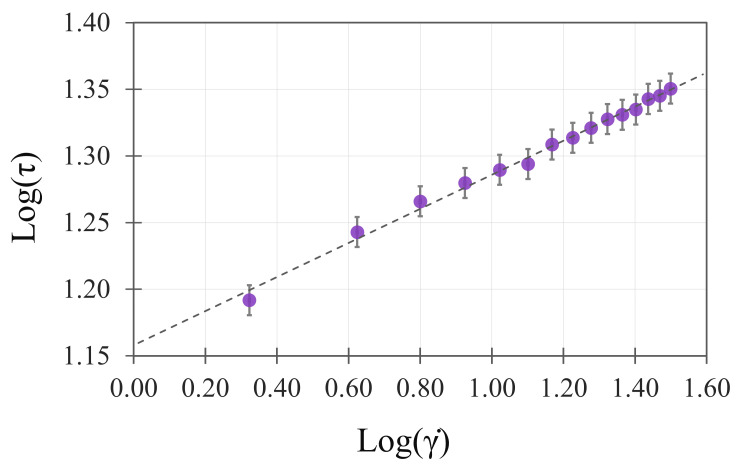
Linear regression between log(γ) and log(τ). Each point means a record from a specific trial, while the error bar indicates the estimated model’s error. The dashed line means the linear regression line.

The consistency evaluation confirmed a typical linear relationship for non-Newtonian fluids between $\dot{\gamma }$ and τ, as shown in Figure 1. The linear regression model yielded a *n* of 0.128, a K of 1.158, and an estimated error of 0.004. The model explained 99.3% of the total variance, as the R-squared value (R^2^) indicated. Furthermore, an ANOVA confirmed the significance of the linearity in the model, with *p*-values < 0.001. Rheological behaviour was consistent with the previously reported pseudoplastic model [44].

**Figure 2:**
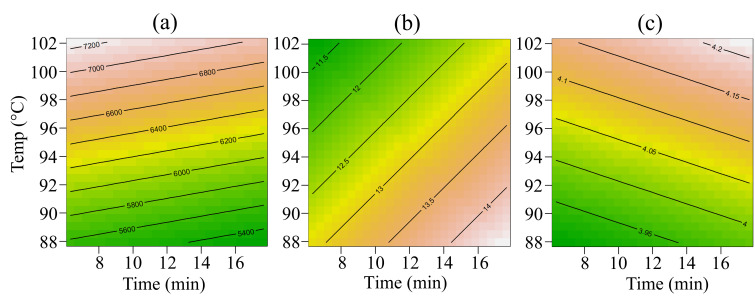
RSM plot according to the two factors: Temp on the vertical axis and Time on the horizontal axis. Green-yellow tones mean lower value, while brown-white tones mean higher value. The charts follow the order: (**a**) Consistency (mPa s), (**b**) syneresis (area percentage), and (**c**) pH.

**Figure 3:**
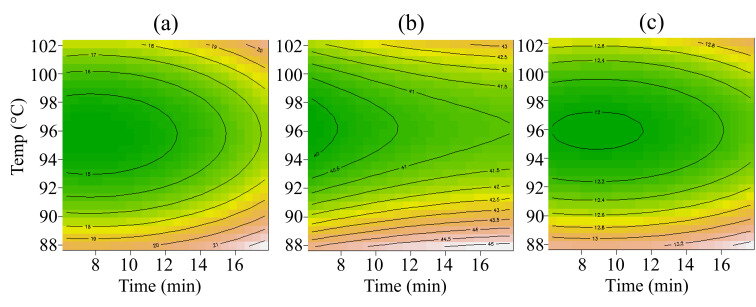
RSM plot according to the two factors: Temp on the vertical axis and Time on the horizontal axis. Green-yellow tones mean lower value, while brown-white tones mean higher value. The charts follow the order: (**a**) ΔE, (**b**) Chroma, and (**c**) BI.

Data collected from all three batches of cooked purées are listed in Table A1 and Table A2. The raw data were joined to compute the RSM on viscosity, syneresis, colour, and pH. These four properties showed a common trend between batches, and the model could fit all the records in terms of *p*-value and not significant lack of fit, as summarised in Table 2. Figure 2 and Figure 3 describe the matrix feature’s response according to the Time and Temp of the thermal treatment. In Figure 2, all models showed a linear trend linked with the Temp factor. In detail, according to Temp, consistency rose, on average, from 5447 mPa s to 8118 mPa s, syneresis decreased, on average, from 13.8% to 11.3%, and pH rose from 3.9 to 4.2 (average data from Table A1). Increasing Time was relevant only for syneresis. Figure 3 describes how purées’ colour behaved according to Temp and Time. Both linear and quadratic Temp effects were found relevant. Medium-high temperature reduced the Chroma, which describes the colour intensity. At the same Temp range, both ΔE and BI retrieved the lowest values. On average, Chroma, ΔE, and BI at 95 °C resulted in 40.9, 15.2, and 12.2, respectively. The same properties generally reached the highest values at temperatures above 100 °C or lower than 90 °C when heated for more than 12 min. At these extremes, Chroma, ΔE, and BI reached values higher than 45.7, 21.1, and 13.2, respectively (average data from Table A2). Finally, a slight batch effect was found on ΔE, the highest average ΔE was recorded on B17 (18.4 ± 3.6) and the lowest on B19 (15.4 ± 2.7).

Regarding the other properties, a pivotal effect is due to the batch of provenance. The batch effect affected the spread of records. Consequently, RSM failed to provide a robust predictive model in these cases. Thus, a specific mixed-effect model was designed for TSSs, WC, PME, PPOs, POs, TPC, and TEAC to find the relationship between the batch’s effect and factors. An ANOVA was performed on models, and the results are presented in Table 3. The ANOVA underlined that TSSs, WC, PPOs, POs, and TEAC varied between batches, while TPC were always comparable. B19 showed the highest WC and relatively lowest TSSs. B17 returned almost absent PPO activity, while B19 had the highest PO activity. Moreover, an interaction effect between batch and Temp was identified for TSSs, while in PE, the interaction was found between batch, Temp and Time. Finally, the batch effect was the only source of variance in WC. Temp and Time were relevant in TSSs. Specifically, the lowest TSSs were recorded in the longest thermal treatment performed at the highest Temp (12 min, 102.1 °C), which measured 9.2 °Brix and 10.4 °Brix for B17 and B19, respectively. On the other hand, the trial performed at the lowest Temp (87.9 °C) recorded TSSs of 14.4 °Brix and 11.2 °Brix for B17 and B19, respectively. Regarding the enzyme residual activity, each enzyme category retrieved a singular response towards the thermal treatment. Details of enzyme residual activity are reassumed in Table 4, grouped per apple batch. Finally, the batch effect was also highlighted in TEAC, where batch B19 demonstrated the highest antioxidant capacity.

## 4. Discussion

The exponential relationship derived from Figure 1 and the *n* value obtained from Equation (7) significantly deviates from 1, confirming that the present purées exhibit non-Newtonian pseudoplastic behaviour, similar to many other fruit purées [45]. Because the *n* value recorded on purée is lower than one, purée can be classified as shear-thinning fluid behaviour. Moreover, the Shear Thinning Index was computed as the ratio between the viscosity at 10 rpm (i.e., 23,427 ± 131 mPa s) and the viscosity at 100 rpm (i.e., 3142 ± 101 mPa s), resulting in 7.5, which is much higher than one [46]. Thus, the speed-dependent consistency typical for this category of fluids was again remarked.

The mechanical and thermal treatments significantly altered the physical properties of the purées. Notably, the consistency of the purées increased substantially with higher temperatures, as supported by recent studies [2,23,47]. This increase in consistency can be primarily attributed to the solubilisation of pectin induced by the cooking temperature [24,48]. Additionally, high cooking Temp (above 95.0 °C) tends to produce smaller particle sizes from the original fruits, leading to a more pronounced deterioration of cell walls. Finally, the pectin solubilisation into the serum (the liquid part of purées) and its gelatinisation rate is notably accelerated by temperature [49], resulting in a higher water binding and, consequently, a higher consistency [8]. These factors’ combined effect plays a crucial role in the Temp and Time directly associated with consistency and syneresis [50,51].

**Figure 4:**
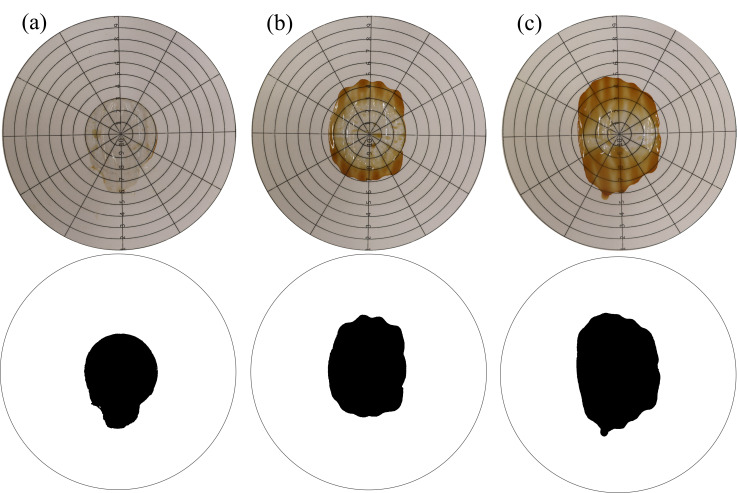
Three examples of outcomes from the visual evaluation of syneresis from three trials of B17. Fluids are spread on paper after 24 h, on the top and on the 2-bit segmented mask on the bottom. Results consisted of the percentage of area occupied by the black spot. The Temp, Time, and related syneresis follow this order: (**a**) 100.0 °C, 8 min, 10.0%, (**b**) 95.0 °C, 12 min, 12.2%, and (**c**) 90.0 °C, 16 min, 16.9%.

Moreover, syneresis assessments confirmed the anticipated outcomes predicted by standard approaches [2,23]. An example of this evaluation is shown in Figure 4. The lowest syneresis was recorded at high temperatures and with shorter thermal treatment durations. In those trials, the temperature was equal to or above 100.0 °C and 8 min or lower. Figure 4a, for instance, shows a very small footprint left by the tested purée, whereas the spot in Figure 4c is noticeably wider compared to Figure 4a,b. The highest viscosity value and the lowest syneresis potential are key factors in meeting common consumer preferences [14,16].

Therefore, achieving the highest temperature while minimising the thermal treatment duration may represent the optimal combination of factors if a dense and compact product is pursued, as usual. On the contrary, middle temperature and long heating duration would produce more liquid purées, which are, on the one hand, less appreciated by consumers but favourable for following operations (i.e., mixing, transporting, or wrapping).

A slight reduction in TSSs was observed with increasing Temp; however, no significant relationship was found between TSSs and viscosity. Although a positive correlation might be expected, the changes in TSSs were likely insufficient to affect consistency [45].

Colour changes after thermal processing of food can be attributed to several factors. Enzymatic oxidation, pigment and molecular alterations, and the Maillard reaction are the primary causes identified in the scientific literature [25,52]. Usually, enzyme activity is carried on until 50−60 °C, reaching the maximum rate at 30−40 °C. Thus, the pre-heating phase is crucial in this context.

**Figure 5:**
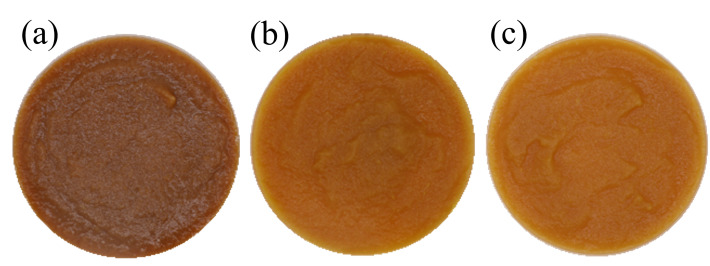
Three examples of apple purée treated at different temperatures for colour evaluation: (**a**) no heating control purée, (**b**) 102.1 °C, and (**c**) 87.9 °C.

An example of colour changes in purées processed from the same batch is shown in Figure 5. Figure 5a represents the control purée, which underwent no thermal treatment and is the darkest and most brown. The colour differences are also distinguishable by the human eye, considering that the average ΔE resulted in 11.5 between cooked and not cooked purées (average data from Table A2). Oxygen exposure and moderate temperature storage (30 min) likely enhanced PPO and PO activity, which are well-documented contributors to enzymatic browning in fruit-based products [11]. Indeed, the brown tonality in Figure 5a is too dark, exceeding any acceptable threshold from a consumer perspective. Despite the Maillard reaction being identified as the primary cause of browning in fruit products undergoing thermal treatment, caramelisation and other reactions may have contributed to the well-known non-enzymatic browning in cooked purées, as the enzymes were almost deactivated after cooking, even at the lowest temperatures [51]. A slight variability of ΔE was detected among trials from the same batch. Notably, differences in ΔE were recorded between 87.9 °C, 95.0 °C, and 102.1 °C. As a consequence, the non-enzymatic browning is supposed to have the primary effect in cooked purées, with temperature being the key driving factor [53]. Therefore, batch B17 showed the overall lowest ΔE and the highest TEAC, while the opposite occurred for batch B19. That proved the natural content of antioxidant compounds might help preserve the final purée’s colour, at least protecting the purée from the enzymatic browning in the initial phases when enzymes are active.

A slight reduction in TSSs was observed with increasing temperature, although WC did not vary significantly between trials. This suggests that the Maillard reaction occurred because some of the TSSs likely reacted in the cooked purées compared to the control ones, with Temp acting as a catalysing factor. The b* value from the CIELab colour space is strongly correlated with browning in fruit-derived foods [26]. On average, the b* value increased from 28 in the control purée to 39 in the heated purées. On the other hand, the highest b* was recorded at 87.0 °C, while the lowest at 100.0 °C (44.0 and 36.5, respectively). The b* value means the yellow-blue balance, in which yellow indicates light tones, which are usually preferable in fruit-based products. Medium-high treatment temperature retrieved the lowest Chroma on purée’s colour. Lower Chroma means dull tint, which is closer to non-treated purées and is related to lower BI values. Thus, the main colour changes affected the intensity, while the overall Hue and L* did not show any detectable trends among the treatments.

Given that high temperatures suppress PPO activity and that non-enzymatic browning reaction products possess antioxidant properties in heated fruit products, the non-enzymatic browning reaction is likely the main cause of colour changes between purées [54,55]. Thus, moderate heating Temp can help preserve purées from enzymatic browning compared to the control. However, excessive heating can intensify the non-enzymatic browning, leading to pronounced browning in the final products, which correlates linearly with temperature [56,57,58]. This relationship is evident in the decreasing trend between ΔE and temperature, as shown in Figure 3.

Contrary but comparable results were found when comparing the colour of thermally treated purées with those that did not undergo any browning process: the lowest temperature preserved the original purée colour mostly [23]. Similar outcomes have also been observed in strawberries by [29]. TPC and TEAC mould the colour of the base product. The polyphenol content was comparable among batches, ensuring an equal colour potential in each apple batch. On the contrary, TEAC is dependent on the batch effect; for example, storage conditions could preserve or deteriorate any antioxidant compounds [59]. TPC and TEAC did not follow any trends with Temp or Time. Indeed, both factors can enhance the release of phenols from vegetable cells and simultaneously deteriorate or alter reactions between phenols and other compounds [60,61].

A unique model for predicting enzyme deactivation during thermal treatment failed due to the prevalence of a batch effect observed. The enzyme activity depends on the apple variety and the specific characteristics of the apples used [62]. Among these characteristics, the ripening level is fundamental [63]. The charts in Figure 4a,b illustrate variations in TSSs and WC, suggesting considerable variability in ripening levels across batches.

A significant decrease in PME activity was observed with increasing heating temperature in batch B18, whereas PME activity remained relatively stable across varying temperatures in batches B17 and B19. This variation led to the interaction effect reported in Table 3. PME residual activity should be nearly deactivated when apples are heated above 59 °C [64]. Strawberries have also demonstrated similar deactivation effects [65]. Adequate PME deactivation is essential for maintaining a desirable purée firmness, which is critical for consumer satisfaction. When properly treated, pectin can absorb water, thereby increasing viscosity and reducing syneresis.

A negative interaction between Temp and Time was found for POs. Therefore, increasing both the Temp and thermal treatment duration reduces residual PO activity. A target temperature of 90.0 °C has been proposed for substantial PO deactivation [13]. Finally, PPO activity remained relatively stable under the conditions tested. However, heating to 80.0 °C, even for a short duration, is sufficient to deactivate most PPO activity [62].

The results indicate that the among-batch stable properties can be consistently predicted based on heating temperature and duration. Conversely, evaluating the raw fruit is essential to managing batch-dependent variables. Assessing apple quality before processing can help predict the risk of enzymatic browning (e.g., by adjusting ascorbic acid dosage or incorporating a steam-blanching step) and to estimate purée sweetness. Future studies should focus on estimating and classifying raw apples using appropriate monitoring techniques to control better the key parameters influencing the physicochemical quality of apple purées.

## 5. Conclusions

Different combinations of temperature and heating duration were tested on three apple purée batches to model and predict their effects on rheology, chemical properties, and enzyme deactivation using Response Surface Methodology. The results showed that consistency, syneresis, pH, Chroma, ΔE, and BI were stable across batches, whereas TSSs, WC, TPC, antioxidant compounds, and enzyme activity were batch-dependent. Stable properties can therefore be predicted through heating parameters, while batch-dependent ones require prior evaluation of raw fruit to achieve the desired purée characteristics. Temperature was the main factor influencing texture, syneresis, and colour. Treatments above 95 °C for less than 12 min improved texture by enhancing consistency and reducing syneresis; however, higher temperatures (i.e., 95–102 °C) intensified browning. Longer heating durations also increased syneresis. The optimal balance between colour preservation and consistency should be determined according to product and consumer requirements. Even the mildest treatment effectively deactivated most enzymes, while natural antioxidants may have contributed to limiting browning.

## Figures and Tables

**Table 1:** Each line indicates a specific trial (purée production), while columns list heating time (Time), the heating temperatures (Temp), and the repetition number selected for the experimental design. The same procedure was replicated in each batch of apples.

Time (min:s)	Temp (°C)	Repetition
12:00	87.9	1
08:00	90.0	1
16:00	90.0	1
12:00	95.0	4
06:20	95.0	1
17:42	95.0	1
08:00	100.0	1
16:00	100.0	1
12:00	102.1	1

**Table 2:** A statistical report of the RSM *models* was obtained on the physical properties of purées, merging data from three batches.

Batch	Variable	Linear			Quadratic			Lack
		Temp	Time	Interaction	Temp	Time	*p*-Value	of Fit
All	Consistency	**	NS	NS	NS	NS	*	NS
All	Syneresis	*	*	NS	NS	NS	*	NS
All	pH	***	NS	NS	NS	NS	**	NS
All	ΔE	*	NS	NS	NS	NS	*	NS
All	Chroma	**	NS	NS	**	NS	*	NS
All	BI	**	NS	NS	**	NS	*	NS

The symbols mean the relevance of variables: “***” *p*-value < 0.001, “**” *p*-value < 0.01, “*” *p*-value < 0.05, while “NS” is non-significant.

**Table 3:** Outcomes from the ANOVA performed on the specific mixed models, including the batch’s effect and the interaction between factors and batch: Total soluble solids (TSSs), Water content (WC), Pectin methylesterase (PME), Polyphenoloxidases (PPOs), and Peroxidases (POs).

Variable	Linear			Quadratic		Batch	Interaction with Batch	
	Temp	Time	Interaction	Temp	Time		Temp	Time
TSSs	**	*	NS	NS	NS	***	*	NS
WC	NS	NS	NS	NS	NS	*	NS	NS
PME	*	NS	NS	NS	NS	NS	**	*
PPOs	NS	NS	NS	NS	NS	*	NS	NS
POs	NS	NS	*	NS	NS	*	NS	NS

The symbols mean the relevance of variables: “***” *p*-value < 0.001, “**” *p*-value < 0.01, “*” *p*-value < 0.05, while “NS” is non-significant.

**Table 4:** Average and standard deviation computed on the residual enzymatic activity per apple batch: pectin methylesterase (PME), peroxidases (POs), polyphenoloxidases (PPOs), total polyphenols (TPC), and the antioxidant capacity (TEAC).

**Variable**	**Unit**	**B17**	**B18**	**B19**
PME	units mL^−1^	2.592 a ± 0.331	2.824 a ± 0.500	3.519 a ± 0.248
POs	ABS min^−1^ g^−1^	0.668 a ± 0.016	1.188 ab ± 0.025	2.079 b ± 0.049
PPOs	ABS min^−1^ g^−1^	0.099 b ± 0.002	0.121 c ± 0.002	0.063 a ± 0.001
TPC	mg kg^−1^	301.6 ± 144.1	323.7± 79.1	272.5 ± 18.6
TEAC	mg kg^−1^	839.7 b ± 149.6	725.2 b ± 137.9	1227.7 a ± 250.5

Different letters “a, b, c” indicate statistical differences among Tukey’s multiple comparisons test at *p* ≤ 0.05.

## Data Availability

The original contributions presented in this study are included in the article. Further inquiries can be directed to the corresponding author .

## Appendix A

**Table A1:** The raw physical and chemical data of every trial are summarised. Control means a purée which did not undergo thermal treatment.

Batch	Time	Temp	Viscosity	Syneresis	TSSs	pH	WC
	(min:s)	(°C)	(mPa s)		(°Brix)		(%)
B17	Control		3312	15.9	11.100	4.1	88.1
B17	08:00	90.0	5764	13.6	13.500	3.8	87.2
B17	16:00	90.0	6088	16.9	13.400	4.1	86.2
B17	08:00	100.0	7420	10.0	13.200	4.3	85.9
B17	16:00	100.0	7272	15.4	10.700	4.2	87.4
B17	12:00	95.0	7296	12.2	13.700	4.3	86.2
B17	12:00	95.0	6052	13.3	13.400	4.0	86.5
B17	06:34	95.0	6128	11.4	13.900	4.2	85.4
B17	17:42	95.0	5420	12.9	12.500	4.2	87.3
B17	12:00	87.9	5190	15.6	14.400	3.8	86.4
B17	12:00	102.1	9266	10.7	9.200	4.3	83.2
B17	12:00	95.0	7220	12.5	13.100	4.1	86.2
B17	12:00	95.0	6763	12.4	14.200	4.1	85.5
B18	Control		4040	15.1	11.5	3.9	87.5
B18	08:00	90.0	6220	13.5	12.600	3.9	85.9
B18	16:00	90.0	6024	13.6	12.200	4.0	86.7
B18	8:00	100.0	5432	11.1	12.700	4.4	86.4
B18	16:00	100.0	5408	13.1	13.100	4.0	85.6
B18	12:00	95.0	6100	12.2	13.200	4.0	86.4
B18	12:00	95.0	5043	11.9	12.300	4.0	87.0
B18	06:34	95.0	7452	12.3	12.300	4.0	86.7
B18	17:42	95.0	4264	12.0	10.200	4.0	85.6
B18	12:00	87.9	5680	12.7	12.500	4.0	85.6
B18	12:00	102.1	5748	11.1	11.300	4.0	86.3
B18	12:00	95.0	6961	12.3	11.500	4.3	85.8
B18	12:00	95.0	6085	12.4	11.600	4.2	84.9
B19	Control		3384	17.5	10.1	3.8	88.7
B19	08:00	90.0	5204	12.2	10.600	3.9	86.7
B19	16:00	90.0	5876	12.4	10.600	4.0	87.4
B19	8:00	100.0	6616	14.9	10.200	4.0	86.8
B19	16:00	100.0	5952	14.6	10.100	4.1	88.3
B19	12:00	95.0	7116	12.4	10.300	4.1	87.8
B19	12:00	95.0	5800	14.6	10.500	4.1	87.6
B19	06:34	95.0	4504	12.2	10.200	3.9	87.3
B19	17:42	95.0	6712	12.8	10.000	4.1	88.0
B19	12:00	87.9	5472	13.0	11.200	4.0	87.3
B19	12:00	102.1	9340	12.1	10.400	4.2	87.1
B19	12:00	95.0	6252	13.0	10.800	4.1	87.9
B19	12:00	95.0	6633	13.9	10.400	3.9	86.2

**Table A2:** The raw colour analysis performed in every trial is resumed. Control means a purée that did not undergo thermal treatment used to compute ΔE. Time is indicated as min:s format, while Temp is in °C.

Batch	Time	Temp	L	a	b	Chroma	Hue	ΔE	BI
B17	Control		46.0	18.0	27.3	34.8	1.0	0.0	12.3
B17	08:00	90.0	57.3	18.3	41.7	45.5	1.2	20.7	13.8
B17	16:00	90.0	59.5	18.0	42.5	46.2	1.2	22.7	13.4
B17	8:00	100.0	54.5	20.5	36.5	41.9	1.1	14.9	12.2
B17	16:00	100.0	58.0	16.0	40.0	43.1	1.2	20.9	12.6
B17	12:00	95.0	54.0	20.0	35.5	40.7	1.1	13.7	12.3
B17	12:00	95.0	59.0	19.0	38.5	42.9	1.1	21.5	12.1
B17	06:34	95.0	55.0	21.5	35.5	41.5	1.0	14.5	12.4
B17	17:42	95.0	57.0	18.0	39.5	43.4	1.1	18.8	13.0
B17	12:00	87.9	58.5	22.0	44.0	49.2	1.1	23.1	12.9
B17	12:00	102.1	55.5	17.5	40.5	44.1	1.2	20.0	12.9
B17	12:00	95.0	56.0	19.0	35.0	39.8	1.1	13.9	11.6
B17	12:00	95.0	56.5	16.0	35.0	38.5	1.1	15.4	12.1
B18	Control		45.0	19.0	29.0	36.4	1.0	0.0	13.6
B18	08:00	90.0	53.5	19.0	36.0	40.7	1.1	13.2	12.8
B18	16:00	90.0	57.5	13.0	38.0	40.2	1.2	17.7	11.6
B18	8:00	100.0	56.0	15.0	37.0	39.9	1.2	16.2	11.9
B18	16:00	100.0	61.0	15.0	39.0	41.8	1.2	20.4	12.2
B18	12:00	95.0	56.0	15.0	38.0	40.9	1.2	16.1	12.1
B18	12:00	95.0	59.5	20.0	43.0	44.4	1.1	20.9	12.8
B18	06:34	95.0	56.0	14.0	36.5	39.1	1.2	16.0	11.5
B18	17:42	95.0	56.0	18.0	40.0	43.9	1.1	17.5	13.5
B18	12:00	87.9	58.0	19.5	40.5	44.9	1.1	19.5	13.2
B18	12:00	102.1	56.0	17.0	37.5	41.2	1.1	14.7	12.9
B18	12:00	95.0	53.5	17.5	32.5	38.9	1.1	10.5	11.1
B18	12:00	95.0	53.5	17.0	34.0	40.0	1.1	11.6	11.7
B19	Control		44.3	17.0	28.0	35.4	1.1	0.0	13.6
B19	08:00	90.0	56.0	16.0	38.0	41.2	1.2	17.1	12.4
B19	16:00	90.0	53.0	17.0	34.5	38.5	1.1	14.1	12.0
B19	8:00	100.0	57.0	14.0	37.0	39.6	1.2	16.4	11.4
B19	16:00	100.0	55.5	17.0	36.0	39.8	1.1	17.0	11.9
B19	12:00	95.0	52.0	16.0	33.5	38.1	1.1	10.8	11.8
B19	12:00	95.0	55.0	16.0	39.0	42.2	1.2	16.1	12.2
B19	06:34	95.0	54.5	15.0	36.0	39.0	1.2	13.4	11.9
B19	17:42	95.0	55.0	17.0	35.5	39.4	1.1	14.0	11.8
B19	12:00	87.9	57.0	13.0	41.0	43.0	1.3	20.6	13.4
B19	12:00	102.1	55.5	19.5	39.0	43.6	1.1	16.9	13.5
B19	12:00	95.0	53.0	17.0	34.0	38.0	1.1	11.1	11.8
B19	12:00	95.0	57.5	19.5	39.0	43.6	1.1	16.9	12.8

## References

[1] Ismea Repot Mercati Agricoli. https://www.ismea.it/istituto-di-servizi-per-il-mercato-agricolo-alimentare.

[2] Buergy A., Rolland-Sabaté A., Leca A., Falourd X., Foucat L., Renard C.M.G.C. (2021). Pectin Degradation Accounts for Apple Tissue Fragmentation during Thermomechanical-Mediated Puree Production. Food Hydrocoll..

[3] Awuah G.B., Ramaswamy H.S., Economides A. (2007). Thermal Processing and Quality: Principles and Overview. Chem. Eng. Process. Process Intensif..

[4] Castaldo D., Laratta B., Loiudice R., Giovane A., Quagliuolo L., Servillo L. (1997). Presence of Residual Pectin Methylesterase Activity in Thermally Stabilized Industrial Fruit Preparations. LWT Food Sci. Technol..

[5] Amiot M.J., Aubert S., Nicolas J. (1993). Phenolic Composition and Browning Susceptibility of Various Apple and Pear Cultivars at Maturity. Acta Hortic..

[6] Nicolas J.J., Richard-Forget F.C., Goupy P.M., Amiot M., Aubert S.Y. (1994). Enzymatic Browning Reactions in Apple and Apple Products. Crit. Rev. Food Sci. Nutr..

[7] Salas-Tovar J.A., Flores-Gallegos A.C., Contreras-Esquivel J.C., Escobedo-García S., Morlett-Chávez J.A., Rodríguez-Herrera R. (2017). Analytical Methods for Pectin Methylesterase Activity Determination: A Review. Food Anal. Methods.

[8] Espinosa-Muñoz L., Renard C.M.G.C., Symoneaux R., Biau N., Cuvelier G. (2013). Structural Parameters That Determine the Rheological Properties of Apple Puree. J. Food Eng..

[9] Mathew A.G., Parpia H.A.B., Chichester C.O., Mrak E.M., Stewart G.F.B.T.-A. (1971). Food Browning as a Polyphenol Reaction. Advances in Food Research.

[10] Loew O., United States Department of Agriculture (1901). Catalase, a New Enzym of General Occurrence with Special Reference to the Tobacco Plant.

[11] Arnold M., Gramza-Michałowska A. (2022). Enzymatic Browning in Apple Products and Its Inhibition Treatments: A Comprehensive Review. Compr. Rev. Food Sci. Food Saf..

[12] Chisari M., Barbagallo R.N., Spagna G. (2007). Characterization of Polyphenol Oxidase and Peroxidase and Influence on Browning of Cold Stored Strawberry Fruit. J. Agric. Food Chem..

[13] Kim A.N., Lee K.Y., Rahman M.S., Kim H.J., Kerr W.L., Choi S.G. (2021). Thermal Treatment of Apple Puree under Oxygen-Free Condition: Effect on Phenolic Compounds, Ascorbic Acid, Antioxidant Activities, Color, and Enzyme Activities. Food Biosci..

[14] Horie F., Kamei M., Nishibe M., Ogawa Y., Tanibuchi M., Gotow N., Oyama-Okubo N., Kohyama K., Kobayakawa T., Kusakabe Y. (2024). Flavor Intensity Is Reduced in Pureed Food: A Study Using Instrumental and Sensory Analyses. Food Qual. Prefer..

[15] Moon K.M., Kwon E.-B., Lee B., Kim C.Y. (2020). Recent Trends in Controlling the Enzymatic Browning of Fruit and Vegetable Products. Molecules.

[16] Pangborn R., Szczesniak A. (2007). Effects of Hydrocolloids on Flavour and Odour Intensities of Aromatic Compounds. J. Texture Stud..

[17] Sepehr A., Zaborowicz M., Gabardi C., Gabardi N., Biada E., Luzzini M., Zanchin A., Guerrini L. (2026). Machine Learning Approach to Inline Monitoring of Apple Puree Consistency through Process Data and Fruit Characteristics. J. Food Eng..

[18] Cánovas J.A., Gea-Botella S., Borrás F., Martí N., Valero M., Saura D., Martínez-Madrid M.C., Laencina J. (2020). Vitamin C Loss Kinetics and Shelf Life Study in Fruit-Based Baby Foods during Post Packaging Storage. Food Packag. Shelf Life.

[19] Pitotti A., Elizalde B.E., Anese M. (1994). Effect of Caramelization and Maillard Reaction Products on Peroxidase. J. Food Biochem..

[20] de Castro B.R., Tribst A.A.L., Cristianini M., Şentürk M. (2017). Effect of High-Pressure Technologies on Enzymes Applied in Food Processing. Enzyme Inhibitors and Activators.

[21] Umair M., Jabbar S., Lin Y., Nasiru M.M., Zhang J., Abid M., Murtaza M.A., Zhao L. (2022). Comparative Study: Thermal and Non-Thermal Treatment on Enzyme Deactivation and Selected Quality Attributes of Fresh Carrot Juice. Int. J. Food Sci. Technol..

[22] Martinez M.V., Whitaker J.R. (1995). The Biochemistry and Control of Enzymatic Browning. Trends Food Sci. Technol..

[23] Guerra L., Romagnoli G., Vignali G. (2012). Extraction of Golden Delicious Apple Puree: Experimental Comparison of Three Different Methods. J. Food Eng..

[24] Buergy A., Rolland-Sabaté A., Leca A., Renard C.M.G.C. (2021). Apple Puree’s Texture Is Independent from Fruit Firmness. LWT.

[25] Maskan M. (2008). Effect of Thermal Processing on Tristimulus Colour Changes of Fruits. Stewart Postharvest Rev..

[26] Hirschler R. (2012). Whiteness, yellowness, and Browning in Food Colorimetry. Color Food Technol. Psychophys. Asp..

[27] Silva F.M., Sims C., Balaban M.O., Silva C.L.M., O’Keefe S. (2000). Kinetics of Flavour and Aroma Changes in Thermally Processed Cupuaçu (*Theobroma grandiflorum*) Pulp. J. Sci. Food Agric..

[28] Castro S.M., Saraiva J.A., Lopes-da-Silva J.A., Delgadillo I., Van Loey A., Smout C., Hendrickx M. (2008). Effect of Thermal Blanching and of High Pressure Treatments on Sweet Green and Red Bell Pepper Fruits (*Capsicum annuum* L.). Food Chem..

[29] Terefe N.S., Matthies K., Simons L., Versteeg C. (2009). Combined High Pressure-Mild Temperature Processing for Optimal Retention of Physical and Nutritional Quality of Strawberries (*Fragaria* × *Ananassa*). Innov. Food Sci. Emerg. Technol..

[30] Chakraborty S., Rao P.S., Mishra H.N. (2015). Effect of Combined High Pressure–Temperature Treatments on Color and Nutritional Quality Attributes of Pineapple (*Ananas comosus* L.) Puree. Innov. Food Sci. Emerg. Technol..

[31] Badin E.E., Rossi Y.E., Montenegro M.A., Ibarz A., Ribotta P.D., Lespinard A.R. (2020). Thermal Processing of Raspberry Pulp: Effect on the Color and Bioactive Compounds. Food Bioprod. Process..

[32] Liaotrakoon W., de Clercq N., van Hoed V., van de Walle D., Lewille B., Dewettinck K. (2013). Impact of Thermal Treatment on Physicochemical, Antioxidative and Rheological Properties of White-Flesh and Red-Flesh Dragon Fruit (*Hylocereus* spp.) Purees. Food Bioprocess Technol..

[33] Salazar-Orbea G.L., García-Villalba R., Tomás-Barberán F.A., Sánchez-Siles L.M. (2021). High–Pressure Processing vs. Thermal Treatment: Effect on the Stability of Polyphenols in Strawberry and Apple Products. Foods.

[34] Wang Z., Bureau S., Jaillais B., Renard C.M.G.C., Chen X., Sun Y., Lv D., Pan L., Lan W. (2024). Infrared Guided Smart Food Formulation: An Innovative Spectral Reconstruction Strategy to Develop Anticipated and Constant Apple Puree Products. Food Innov. Adv..

[35] Espinosa L., To N., Symoneaux R., Renard C.M.G.C., Biau N., Cuvelier G. (2011). Effect of Processing on Rheological, Structural and Sensory Properties of Apple Puree. Procedia Food Sci..

[36] Szczepańska J., Pinto C.A., Skąpska S., Saraiva J.A., Marszałek K. (2021). Effect of Static and Multi-Pulsed High Pressure Processing on the Rheological Properties, Microbial and Physicochemical Quality, and Antioxidant Potential of Apple Juice during Refrigerated Storage. LWT.

[37] El Bouchikhi S., Pagès P., El Alaoui Y., Ibrahimi A., Bensouda Y. (2019). Syneresis Investigations of Lacto-Fermented Sodium Caseinate in a Mixed Model System. BMC Biotechnol..

[38] Heydari S., Amiri-Rigi A., Ehsani M.R., Mohammadifar M.A., Khorshidian N., Koushki M.R., Mortazavian A.M. (2018). Rheological Behaviour, Sensory Properties and Syneresis of Probiotic Yoghurt Supplemented with Various Prebiotics. Int. J. Dairy Technol..

[39] Kertesz Z.I. (1937). Pectic Enzymes: I. The Determination of Pectin-Methoxylase Activity. J. Biol. Chem..

[40] Lee M., Macmillan J.D. (1968). Mode of Action of Pectic Enzymes. I. Purification and Certain Properties of Tomato Pectinesterase. Biochemistry.

[41] Ünal M.Ü., Şener A. (2015). Extraction and Characterization of Pectin Methylesterase from Alyanak Apricot (*Prunus armeniaca* L). J. Food Sci. Technol..

[42] Giusti M.M., Wrolstad R.E. (2001). Characterization and Measurement of Anthocyanins by UV-Visible Spectroscopy. Curr. Protoc. Food Anal. Chem..

[43] Sridhar A., Ponnuchamy M., Kumar P.S., Kapoor A., Vo D.-V.N., Prabhakar S. (2021). Techniques and Modeling of Polyphenol Extraction from Food: A Review. Environ. Chem. Lett..

[44] Kersten E., Barry A., Klein S. (2016). Physicochemical Characterisation of Fluids and Soft Foods Frequently Mixed with Oral Drug Formulations Prior to Administration to Children. Pharmazie.

[45] Maceiras R., Álvarez E., Cancela M.A. (2007). Rheological Properties of Fruit Purees: Effect of Cooking. J. Food Eng..

[46] (2022). Standard Test Method for Shear Thinning Index of Non-Newtonian Liquids Using a Rotational Viscometer.

[47] Saravacos G.D. (1970). Effect of Temperature on Viscosity of Fruit Juices and Purees. J. Food Sci..

[48] Sila D.N., Van Buggenhout S., Duvetter T., Fraeye I., De Roeck A., Van Loey A., Hendrickx M. (2009). Pectins in Processed Fruits and Vegetables: Part II—Structure–Function Relationships. Compr. Rev. Food Sci. Food Saf..

[49] da Silva J.A.L., Gonçalves M.P., Rao M.A. (1995). Kinetics and Thermal Behaviour of the Structure Formation Process in HMP/Sucrose Gelation. Int. J. Biol. Macromol..

[50] Guillon F., Barry J.L., Thibault J.-F. (1992). Effect of Autoclaving Sugar-Beet Fibre on Its Physico-Chemical Properties and Its in-Vitro Degradation by Human Faecal Bacteria. J. Sci. Food Agric..

[51] Müller S., Kunzek H. (1998). Material Properties of Processed Fruit and Vegetables I. Effect of Extraction and Thermal Treatment on Apple Parenchyma. Eur. Food Res. Technol..

[52] Vikram V.B., Ramesh M.N., Prapulla S.G. (2005). Thermal Degradation Kinetics of Nutrients in Orange Juice Heated by Electromagnetic and Conventional Methods. J. Food Eng..

[53] Ibarz A., PagaÂn J., Garza S. (2000). Kinetic Models of Non-Enzymatic Browning in Apple Puree. J. Sci. Food Agric..

[54] Ciou J.-Y., Lin H.-H., Chiang P.-Y., Wang C.-C., Charles A.L. (2011). The Role of Polyphenol Oxidase and Peroxidase in the Browning of Water Caltrop Pericarp during Heat Treatment. Food Chem..

[55] Yilmaz Y., Toledo R. (2005). Antioxidant Activity of Water-Soluble Maillard Reaction Products. Food Chem..

[56] Barreiro J.A., Milano M., Sandoval A.J. (1997). Kinetics of Colour Change of Double Concentrated Tomato Paste during Thermal Treatment. J. Food Eng..

[57] Ávila I.M.L.B., Silva C.L.M. (1999). Modelling Kinetics of Thermal Degradation of Colour in Peach Puree. J. Food Eng..

[58] Rhim J.W., Nunes R.V., Jones V.A., Swartzel K.R. (1989). Kinetics of Color Change of Grape Juice Generated Using Linearly Increasing Temperature. J. Food Sci. Technol..

[59] Arora B., Sethi S., Joshi A., Sagar V.R., Sharma R.R. (2018). Antioxidant Degradation Kinetics in Apples. J. Food Sci. Technol..

[60] Arfaoui L. (2021). Dietary Plant Polyphenols: Effects of Food Processing on Their Content and Bioavailability. Molecules.

[61] ElGamal R., Song C., Rayan A.M., Liu C., Al-Rejaie S., ElMasry G. (2023). Thermal Degradation of Bioactive Compounds during Drying Process of Horticultural and Agronomic Products: A Comprehensive Overview. Agronomy.

[62] Nogales-Delgado S. (2021). Polyphenoloxidase (PPO): Effect, Current Determination and Inhibition Treatments in Fresh-Cut Produce. Appl. Sci..

[63] Coseteng M.Y., Lee C.Y. (1987). Changes in Apple Polyphenoloxidase and Polyphenol Concentrations in Relation to Degree of Browning. J. Food Sci..

[64] Denès J.-M., Baron A., Drilleau J.-F. (2000). Purification, Properties and Heat Inactivation of Pectin Methylesterase from Apple (Cv Golden Delicious). J. Sci. Food Agric..

[65] Bodelón O.G., Avizcuri J.-M., Fernández-Zurbano P., Dizy M., Préstamo G. (2013). Pressurization and Cold Storage of Strawberry Purée: Colour, Anthocyanins, Ascorbic Acid and Pectin Methylesterase. LWT—Food Sci. Technol..

